# Modified Carbapenem Inactivation Method and Ethylenediaminetetraacetic Acid (EDTA)-Carbapenem Inactivation Method for Detection of Carbapenemase-Producing Enterobacterales and Pseudomonas aeruginosa

**DOI:** 10.7759/cureus.63340

**Published:** 2024-06-27

**Authors:** Gaurav Verma, Nipa Singh, Shradha Smriti, Subhra Snigdha Panda, Dipti Pattnaik, Sukanta Tripathy, Ashok K Praharaj, A. Raj Kumar Patro

**Affiliations:** 1 Microbiology, Kalinga Institute of Medical Sciences, Bhubaneswar, IND; 2 Transfusion Medicine, Kalinga Institute of Medical Sciences, Bhubaneswar, IND

**Keywords:** serine-beta lactamase, metallo-β-lactamase, phenotypic test, ecim, mcim, carbapenem-resistant

## Abstract

Introduction: The rising incidence of carbapenem resistance in Enterobacterales and *Pseudomonas aeruginosa* is a concern. Since carbapenemase production is the primary resistance mechanism, detecting and identifying the genes responsible for it is crucial to effectively monitor its spread.

Objective: This study aims to detect positivity for the modified carbapenem inactivation method (mCIM) and ethylenediaminetetraacetic acid (EDTA)-carbapenem inactivation method (eCIM) for the detection of carbapenemase-producing Enterobacterales and *Pseudomonas aeruginosa*.

Methods: Methods: A cross-sectional study was carried out at a tertiary care hospital, including 250 clinical isolates of Enterobacterales and *Pseudomonas aeruginosa*. These isolates exhibited resistance to at least one of the carbapenems as determined by the VITEK AST 2 System (bioMérieux, USA). The isolates were subjected to mCIM testing, and those that tested positive were further tested using eCIM. The results were interpreted in accordance with the guidelines provided by the Clinical and Laboratory Standards Institute (CLSI) 2023.

Results: Out of the total 250 carbapenem-resistant Enterobacterales and *Pseudomonas aeruginosa* isolates, 151 (60.4%) were *Klebsiella pneumonia, *44 (17.6%) were *Escherichia coli,* 10 (4.0%) were *Enterobacter cloacae*, 6 (2.4%) were *Providencia spp*., 4 (1.6%) were *Serratia marcescens*, 4 (1.6%) were *Proteus mirabilis* and 31 (12.4%) were *Pseudomonas aeruginosa*. Positivity for the mCIM was observed in 96% (240 out of 250) of the isolates. Of the mCIM-positive isolates, 234 (97.5%) also tested positive for eCIM, indicating metallo-β-Lactamase (MLB) production. A statistically significant association was found between both mCIM and eCIM positivity and the degree of resistance to carbapenem (p<0.05)*.*

Conclusion: This study shows that the inexpensive method, a combination of mCIM and eCIM assists in differentiating between serine carbapenemase producers and MLB producers, thereby guiding the selection of appropriate therapy and useful in infection control in resource-limited settings.

## Introduction

Antibiotic-resistant bacteria are emerging as a significant public health issue worldwide, leading to recurring patient infections. These drug-resistant microorganisms cause infections in hospitalized patients, limiting the available treatment options and resulting in higher infection and mortality rates [1]. Identifying these drug-resistant organisms promptly is essential to ensure patients receive timely and effective treatment [2].

Carbapenems, including meropenem, imipenem, ertapenem, and doripenem, are effective in treating multi-drug-resistant (MDR) infections. However, the incidence of carbapenem resistance (CR) has increased over the last few decades. CR is commonly observed in Enterobacterales such as *Escherichia coli *and *Klebsiella pneumonia*, as well as in *Pseudomonas aeruginosa* and Acinetobacter spp. [3,4].

According to the Centers for Disease Control and Prevention (Atlanta, Georgia, USA), a carbapenem-resistant organism is an organism that is resistant to one or more carbapenem antibiotics or produces the carbapenemase enzyme [5]. The development of carbapenem resistance is associated with the production of carbapenem-hydrolyzing β-lactamases and due to the production of extended-spectrum beta-lactamases (ESBL). 

Molecular methods are considered the gold standard for detecting carbapenemase enzyme production. However, these methods can be expensive and are typically available only in larger facilities. To make detection more cost-effective, several phenotypic assays have been developed. These include the Combined Disk Test (CDT), modified Hodge Test (MHT), Double Disk Synergy Test (DDST), modified carbapenem inactivation method (mCIM), ethylenediaminetetraacetic acid (EDTA)-carbapenem inactivation method (eCIM), and Carba NP test. The MHT was the first phenotypic test recommended by the Clinical and Laboratory Standards Institute (CLSI) in 2009 for detecting carbapenemase production with good sensitivity and specificity. According to current Clinical and Laboratory Standards Institute guidelines, carbapenemase production detection is performed using three phenotypic tests: mCIM, eCIM, and Carba [6]. The mCIM and eCIM tests could differentiate between serine beta-lactamases and metallo-beta-lactamases, which is useful in selecting therapeutic interventions and infection control.

This study aims to detect carbapenemase production in Enterobacterales and *Pseudomonas aeruginosa* using a combination of the mCIM and eCIM phenotypic methods. A part of this study was presented at MICROCON 2023 National Conference, Indian Association of Medical Microbiologists, held between 23rd and 26th November 2023, at the Scientific Convocation Center, King George's Medical University, Lucknow, India.

## Materials and methods

This cross-sectional study was conducted from May 2023 to September 2023 in the Department of Microbiology at the Kalinga Institute of Medical Sciences, Bhubaneswar, Odisha, India. The study included isolates obtained from various clinical specimens (urine, blood, body fluids, pus, and sputum) routinely received in the microbiology department. A total of 250 clinical isolates were included in the study, comprising Enterobacterales (219 out of 250) and *Pseudomonas aeruginosa* (31 out of 250). These isolates were resistant to at least one of the carbapenems, as per the Clinical and Laboratory Standards Institute (CLSI) guidelines. The study was approved by the Institutional Ethics Committee (reference no. KIIT/KIMS/IEC/1344/2023). Some of the clinical isolates of Enterobacterales and *Pseudomonas aeruginosa* were not resistant to carbapenems and such isolates were excluded from the study.

Procedure

Primary isolates were stored in tryptic soya broth to prevent changes in bacterial properties. The identification and antibiotic sensitivity tests of these clinical isolates were conducted in a microbiology laboratory. These isolates were found to be resistant to at least one of the carbapenems (meropenem, imipenem, or ertapenem), as determined by the VITEK AST 2 System (bioMérieux, USA) using Antimicrobial Susceptibility Testing (AST) panels 405 and 406.

Modified carbapenem inactivation method (mCIM): mCIM was performed to detect the production of the carbapenemase enzyme in Enterobacterales and *Pseudomonas aeruginosa* [6]. EDTA-carbapenem inactivation method (eCIM): When the mCIM test yielded a positive result, the eCIM was then conducted to distinguish between the metallo-β-lactamase (MBL) and serine-β-Lactamase enzymes [6].

Modified carbapenem inactivation method in conjugation with the EDTA-carbapenem inactivation method: The mCIM and eCIM methods were employed to detect CPE, following the CLSI-2023 guidelines. For each strain under test, two tubes, each containing 2 mL of Trypticase Soy Broth (TSB), were used concurrently. One tube was supplemented with 20 µL of 0.5 M EDTA (Sigma), while the other tube was kept EDTA-free. A fresh colony from the strain under test was transferred to each tube using a 1 µL inoculating loop. A 10-mg meropenem disk (HiMedia, India) was then incubated with the bacterial suspension from the tested strain for 2-4 hours at 35°C. Following this, meropenem disks from both tubes were placed on Mueller Hinton agar plates, which were then inoculated with the *E. coli *ATCC 25922 indicator strain. The mCIM was considered to have yielded a positive result when the diameter of the inhibition zone was between 6 and 15 mm, or between 16 and 18 mm but with small colonies present within the inhibitory zone. Interpretation of eCIM results should be undertaken only when the mCIM result indicates the presence of a carbapenemase. If the zone diameter for eCIM exceeds that of mCIM by 5 mm, it suggests a potential production of metallo-carbapenemase. *E. coli* ATCC 25922 was used as a quality control measure.

According to the data provided by CLSI, the mCIM's sensitivity and specificity are greater than 99% for Enterobacterales and range from 97-100% for *Pseudomonas aeruginosa*. In contrast, the eCIM method demonstrates sensitivity and specificity greater than 95% and 92%, respectively, for Enterobacterales. In this study, we conducted mCIM on all 250 isolates and performed eCIM on those isolates that tested positive for mCIM.

Statistical analysis

The statistical analysis was conducted using GraphPad Prism software (version 10) (GraphPad Software, Boston, MA). The association between the carbapenem results, as determined by Vitek MIC, mCIM, and eCIM, was compared using a 2x2 Fisher’s exact test. A p-value of less than 0.05 was considered statistically significant.

## Results

**Figure 1:**
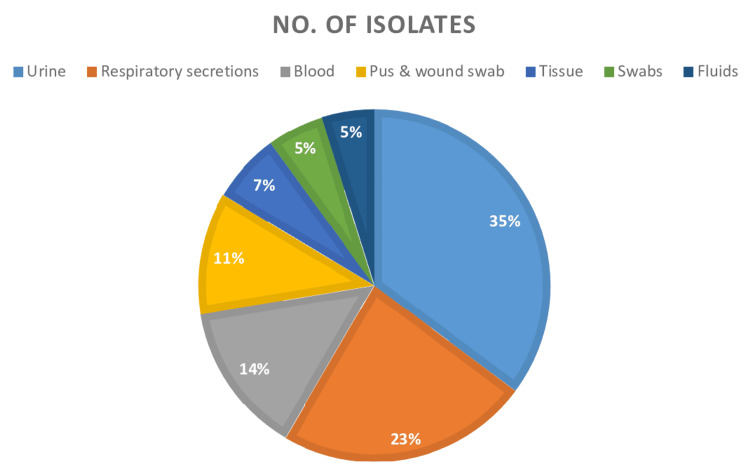
Sample-wise distribution of carbapenem-resistant isolates

Out of the 250 isolates analyzed, 219 (87.6%) were classified as part of the Enterobacterales family, and 31 (12.4%) were identified as *Pseudomonas aeruginosa* isolates. The majority of carbapenem-resistant isolates (61.2%) were from males, accounting for 153 isolates, while 97 (38.8%) isolates were from females. Patients over 60 years of age made up 134 (53.6%) of the 250 cases. The most common source of isolates was urine, with 88 instances (35%) (Figure 1). The majority of isolates were obtained from the ICU, accounting for 153 (61.2%), followed by in-patient wards with 101 (40.4%), and the OPD with 29 (11.6%).

We analyzed samples across seven categories. The most predominant carbapenem-resistant organism was *Klebsiella pneumonia*, accounting for 151 (60.4%) of the cases, followed by *E. coli* with 44 (17.6%) and *Pseudomonas aeruginosa* with 31 (12.4%). Whereas 145 (96%) of *Klebsiella pneumonia*, 41 (93%) of *E. coli*, and 31 (100%) of *Pseudomonas aeruginosa* tested positive for mCIM, suggesting the presence of carbapenemase enzyme production in the isolates.

**Figure 2:**
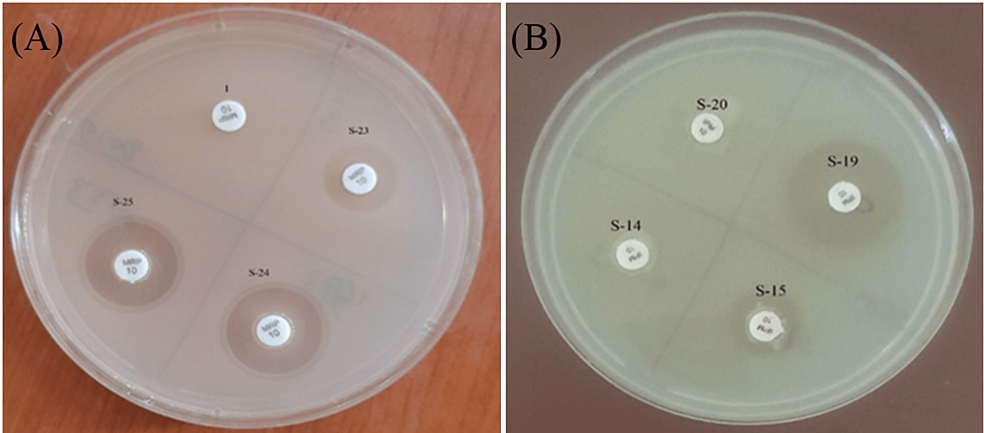
mCIM and eCIM test setup (A) Modified carbapenem inactivation method (mCIM) test: Isolate 1: Positive Control; Isolate 23, 24 & 25: Carbapenemase +ve. (B). Ethylenediaminetetraacetic acid (EDTA)-carbapenem inactivation method (eCIM) test: Isolate 14 & 20: Serine positive; Isolate 15 & 19: Metallo-β-lactamases (MBL) positive.

The mCIM positivity among Enterobacterales ranges from 75% to 100%. Specifically, 145 (96%) of *Klebsiella pneumonia* and 41 (93%) of *E. coli* showed positive results in the mCIM test. Pseudomonas aeruginosa demonstrated a 31 (100%) positive result on the mCIM test (Figure 2), suggesting that a significant proportion of carbapenem resistance is attributable to the production of the carbapenemase enzyme.

We subjected all mCIM-positive isolates to the EDTA-carbapenem inactivation method (eCIM) test. Out of a total of 240 isolates, which included 219 (87.6%) Enterobacterales and 31 (12.4%) *Pseudomonas aeruginosa*, the eCIM test was positive for 142 (98%) of *Klebsiella pneumonia*, 39 (95%) of *E. coli* isolates, and 31 (100%) of *Pseudomonas aeruginosa* isolates. Consequently, 234 (93.6%) carbapenem-resistant Gram-negative bacilli (CRGNB) isolates were eCIM-positive, indicating that 234 (97.5%) were MBL producers and six (2.5%) were serine-β-lactamase producers.

The distribution of carbapenemase in CRGNB varied from 91.4% to 100% across different samples. Among the three most common samples, carbapenemase production was most prevalent in urine samples 85 (96.6%), followed by respiratory secretions 56 (96.5%), and blood 32 (91.4%) (Table 1).

**Table 1:** Distribution of carbapenemase producer samples analysis (positivity to mCIM and eCIM) mCIM: Modified carbapenem inactivation method; eCIM: Ethylenediaminetetraacetic acid (EDTA)-carbapenem inactivation method; CRGNB: Carbapenem-resistant Gram-negative bacilli.

Sample	No. of CRGNB	No. of mCIM +ve isolates N (%)	No. of eCIM +ve isolates N (%)
Urine	88	85 (96.6)	85 (96.5)
Respiratory secretions	58	56 (96.5)	55 (94.8)
Blood	35	32 (91.4)	32 (91.4)
Pus & wound swab	28	27 (96.4)	25 (89.2)
Tissue	16	16 (100)	15 (93.7)
Swabs	13	13 (100)	11 (84.6)
Fluids	12	11 (91.6)	11 (91.6)
Total	250	240 (96.0)	234 (93.6)

Out of the 250 isolates, 207 (82.8%) demonstrated resistance to all carbapenems tested using the automated VITEK AST 2 system. However, 24 (9.6%) isolates displayed either sensitivity or moderate susceptibility to one or more of the tested carbapenems. Notably, meropenem and imipenem were the drugs most frequently showing moderate susceptibility, with an MIC value of 2 µg/mL for 22 (9.4%) isolates (Table 2).

**Table 2:** Carbapenem susceptibility test results (as per VITEK AST 2) VITEK AST 2 System (bioMérieux, USA); AST: Antimicrobial susceptibility testing.

Antibiotic	Susceptible N (%)	Moderately Susceptible N (%)	Resistant N (%)	Total
Imipenem	15 (6.0)	9 (3.6)	226 (90.4)	250
Meropenem	18 (7.2)	12 (4.8)	220 (88.0)	250
Ertapenem *	3 (1.2)	1 (0.4)	207 (82.8)	211*

Table 3 presents an analysis comparing the results from the VITEK AST 2 system and the mCIM test. A 2x2 Fisher’s exact test was performed to investigate the relationship between carbapenem susceptibility testing using the automated minimum inhibitory concentration (MIC) method and mCIM positivity. The analysis revealed a statistically significant correlation between the variables, with a p-value of 0.0027. It was observed that isolates showing resistance to all three carbapenems through MIC determination are more likely to be carbapenemase producers, compared to isolates that are sensitive or moderately sensitive to one or more carbapenems.

**Table 3:** Investigation of the pattern of susceptibility to carbapenems in relation to the positivity of mCIM and eCIM There is a statistically significant association of modified carbapenem inactivation method (mCIM) positivity with the degree of resistance to carbapenem p-value: 0.0027; There is a statistically significant association of ethylenediaminetetraacetic acid (EDTA)-carbapenem inactivation method (eCIM) positivity with the degree of resistance to carbapenem p-value: 0.0001.

Variables	Isolates sensitive or moderately sensitive to at least one of the carbapenem	Isolates resistant to all carbapenem	Total	Chi-square	p-value
mCIM +ve	55	185	240		
mCIM-ve	7	3	10	11.413	0.0027
Total	62	188	250		
Variables	Isolates sensitive or moderately sensitive to at least one of the carbapenem	Isolates resistant to all carbapenem	Total	Chi-square	p-value
eCIM +ve	22	212	234		
eCIM-ve	5	1	6	32.0255	0.0001
Total	27	213	240		

Out of 240 mCIM-positive isolates, 234 (97.5%) were eCIM-positive, indicating the presence of metallo-β-lactamase genes (such as *NDM*, *IMP*, *VIM*, *KPC*, and *IMI*). The remaining six isolates could be serine β-lactamases, or there might be a co-occurrence of serine β-lactamases with *MBL* genes. Among the 234 eCIM-positive isolates, 22 (9.4%) were sensitive or moderately sensitive to at least one of the carbapenems.

The analysis between mCIM and eCIM results is presented in Table 3. A 2x2 Fisher’s exact test was performed to examine the association between mCIM positivity and eCIM. The correlation between these variables showed statistical significance, with a p-value of 0.0001.

## Discussion

Carbapenem, once a highly effective drug for treating Gram-negative infections, has become less effective in treating serious infections caused by Gram-negative bacteria. This is due to these organisms acquiring carbapenem resistance genes, primarily *NDM*, *KPC*, *OXA-48*, *VIM*, and *IMP*. Carbapenem-hydrolyzing β-lactamases are divided into serine-β-lactamases Class A and Class D, which include blaKPC, blaIMI, blaOXA-48, blaOXA-181, blaOXA-23, blaOXA-40, and blaOXA-58. Metallo-β-lactamases of Class B include blaIMP, blaVIM, and blaNDM. In Western countries, the most widespread variety is blaKPC, followed by blaOXA. Previous studies have shown that the most common categories in the Asian subcontinent were MBL, namely blaNDM-1 and blaOXA-48 [7].

In our study, the majority of isolates were obtained from the ICU, accounting for 153 (61.2%), followed by in-patient wards with 101 (27.2%), and the OPD with 29 (11.6%). This distribution is similar to the findings of a study by Baskaran et al. [7]. Our study found that the prevalence of carbapenemase production among the carbapenem-resistant isolates screened by the VITEK AST 2 System was 96% (95% CI: 92.77-98.07). This was particularly true for metallo β-lactamases, with 96% mCIM positivity (i.e., 234 out of 240 were positive for EDTA-carbapenem inactivation method (eCIM). This is consistent with a study by Gallego et al., which showed that the prevalence of mCIM was between 96% and 98% [8]. A study in Lucknow by Rahman et al. recorded a 100% prevalence of *NDM* genes in all the carbapenem-resistant Enterobacterales [9]. According to a study conducted by Roopashree et al., the mCIM method revealed a prevalence rate of 45.09% [10].

Research conducted in the Coimbatore region of Tamil Nadu revealed a prevalence rate of 73.33% between 2011 and 2015 and 82% in 2017 [11]. The majority of these studies focused on isolates from samples taken from patients with specific co-morbid conditions. Furthermore, a study by Gromski et al. found a higher prevalence of positive bile culture in patients who had not previously undergone biliary stent placement [12]. The results showed that 86.1% of patients without a prior biliary stent had positive bile cultures, compared to only 55% of patients in an earlier study who had a positive bile culture without a previous biliary stent. However, some studies were limited to the detection of a specific group of genes responsible for carbapenemases using PCR [11-14].

In our study, the combined prevalence of mCIM and EDTA-carbapenem inactivation method (eCIM) was 97.5%, which aligns with the study by Li and co-workers have reported that the combined incidence of mCIM with eCIM and CDT was 97.5% and 96.2%, respectively [15]. Similarly, Koul et al. stated that the combined prevalence of mCIM and eCIM was 58.5% and 58.4%, respectively [16].

Males constituted 61.2% and females constituted 38.8% in this study. This is similar to the study by Jaiswal et al., which showed a male predominance of 61% [17]. Our study revealed that the three most frequent samples were urine (35%), respiratory secretions (23%), and blood (14%) followed by pus and wound swabs (11%), tissue (7%), swabs (5%), and body fluids (5%) (Figure 1). The highest prevalence of Carbapenem Resistant Enterobacteriaceae (CRE) (32.4%) was found in urine samples, followed by respiratory secretions (CRE-20%) and blood (CRE-14%). In contrast, Jaiswal et al. found urine to be the most common sample (29.33%), but the highest prevalence of CRE was in blood samples [17].

Among the carbapenem-resistant Enterobacterales, *Klebsiella pneumonia* and *E. coli* are the top two carbapenem-resistant organisms, accounting for 60.4% and 17.6%, respectively. This is confirmed by various studies, such as the one by Gao et al., which showed that *Klebsiella pneumonia* was the most common CRE organism (44.8%), followed by *E. coli *(25.8%), and *Enterobacter cloacia* (13.8%) [18].

In this study, Pseudomonas aeruginosa was found to be most prevalent in respiratory secretions, followed by urine, and pus/wound swabs, with prevalence rates of 25.8%, 22.5%, and 22.5%, respectively. These findings are similar to the study by Khater et al., which reported prevalence rates of 37.2% in urine, 30.2% in sputum, 25.6% in pus samples, and 4.66% in blood [19]. In our study, the prevalence of carbapenem-resistant *Pseudomonas aeruginosa* was found to be 12.4%. This is comparable to the study by Cheemala et al., which reported a carbapenem resistance rate of 24% in *Pseudomonas aeruginosa* [20]. Another study by Vamsi et al. reported a carbapenem resistance rate of 10.9% in Pseudomonas spp [21].

According to a report from the Antimicrobial Resistance (AMR) Surveillance Network of India, the prevalence of carbapenem resistance in Enterobacterales was 40%, and the average number of *MBL* genes in these isolates was 30%. The occurrence of NDM was relatively high in certain parts of the country. The AMR Surveillance Network also reported that the increase in the prevalence of resistance in *E. coli* and *Klebsiella pneumonia* was 5% and 17%, respectively, over a five-year period in India (2017-2022) [22].

This study has many limitations and challenges. We have used a combination of mCIM and eCIM for phenotypic detection of carbapenem-producing Enterobacterales and *Pseudomonas aeruginosa*. Further molecular detection of serine beta-lactamases genes and metallo beta-lactamases by real-time PCR would have been useful in discriminating specific enzymes However, we could not differentiate between the co-occurrence of genes with MBL genes and serine ß-lactamases. However, this will not impact the broad findings of the study regarding the use of eCIM and mCIM to detect and distinguish carbapenemase in resource-limited settings.

## Conclusions

The prevalence of infections caused by Gram-negative, carbapenem-resistant pathogens is increasing at an alarming rate. With its high sensitivity and specificity, the mCIM proves to be an effective test for detecting these pathogens. However, for the mCIM to yield consistent and accurate results, it must be conducted as a phenotypic test under standardized conditions, as described by the CLSI guideline. The eCIM test shows a positive result irrespective of the presence or absence of the serine carbapenemase genes in combination with metallo-β-lactamase (MBL). Therefore, this method does not distinguish between the co-occurrence and presence of *MBL* genes in combination with carbapenemases and those of MBL genes alone. The significant occurrence of MBL in eCIM cases implies that the ideal combination for mCIM-positive isolates in this study region is ceftazidime-avibactam plus aztreonam. Implementing phenotypic methods (eCIM and mCIM) to detect and distinguish carbapenemases would be useful in guiding empirical antibiotic therapy, especially in and useful in infection control in resource-limited settings.

## References

[1] Silva DD, Rampelotto RF, Lorenzoni VV, Santos SO, Damer J, Hörner M, Hörner R (2017). Phenotypic methods for screening carbapenem-resistant Enterobacteriaceae and assessment of their antimicrobial susceptibility profile. Rev Soc Bras Med Trop.

[2] Giri S, Sen S, Lall M (2021). Descriptive study for detection of carbapenem resistant enterobacteriaceae by the modified carbapenem inactivation method in a tertiary care hospital of Western Maharashtra. Journal of Evidence Based Medicine and Healthcare.

[3] Verma G, Nayak SR, Jena S (2023). Prevalence of carbapenem-resistant enterobacterales, acinetobacter baumannii, and pseudomonas aeruginosa in a tertiary care hospital in Eastern India: A pilot study. J Pure Appl Microbiol.

[4] (2024). Antimicrobial Resistance Research & Surveillance Network-Annual report January 26, 2022. https://main.icmr.nic.in/sites/default/files/upload_documents/AMRSN_Annual_Report_2022.pdf.

[5] (2024). About Division of Healthcare Quality Promotion. https://www.cdc.gov/ncezid/dhqp/index.ht.

[6] (2024). CLSI M100: Performance Standards for Antimicrobial Susceptibility Testing, 34th Edition. Wayne PA: Clinical and Laboratory Standards Institute.

[7] Baskaran V, Jeyamani L, Thangaraju D, Sivaraj R, Jayarajan J (2023). Detection of carbapenemase production by modified carbapenem inactivation method among carbapenem resistant Gram-negative bacilli at a tertiary care centre in Coimbatore, Tamil Nadu, India. National J of Lab Med.

[8] Gallego M, Salazar-Ospina L, Jiménez JN (2022). The modified carbapenem inactivation method (mCIM): highly sensitive and specific tool to assess carbapenemase producing and non-producing in Gram-negative bacilli (Article in Spanish). Hechos Microbiológicos.

[9] Rahman M, Shukla SK, Prasad KN (2014). Prevalence and molecular characterisation of New Delhi metallo-β-lactamases NDM-1, NDM-5, NDM-6 and NDM-7 in multidrug-resistant Enterobacteriaceae from India. Int J Antimicrob Agents.

[10] Roopashree S, Kaup S Prevalence of various Beta-lactamases in Enterobacteriaceae in a tertiary care hospital in South India: A Cross-sectional study. Int J Med Microbiol Tropical Dis.

[11] Kumar AP, Vinod KS (2017). Detection of carbapenem resistance encoding genes among Gram negative bacteria from urinary tract infection in patients with type 2 diabetes mellitus. J Pure Appl Microbiol.

[12] Gromski MA, Gutta A, Lehman GA (2022). Microbiology of bile aspirates obtained at ERCP in patients with suspected acute cholangitis. Endoscopy.

[13] Isik A, Poyanli A, Tekant Y, Cagatay A, Acunas B, Ibis C, Ozden I (2021). Incomplete or inappropriate endoscopic and radiologic interventions as leading causes of cholangitis. Pol Przegl Chir.

[14] Mulvey MR, Grant JM, Plewes K, Roscoe D, Boyd DA (2011). New Delhi metallo-β-lactamase in Klebsiella pneumoniae and Escherichia coli, Canada. Emerg Infect Dis.

[15] Li J, Li C, Cai X (2019). Performance of modified carbapenem inactivation method and inhibitor-based combined disk test in the detection and distinguishing of carbapenemase producing Enterobacteriaceae. Ann Transl Med.

[16] Koul N, Kakati B, Agarwal S (2022). Use of the combined modified carbapenem inactivation method and EDTA-modified carbapenem inactivation method for detection of carbapenemase-producing Enterobacteriaceae causing ventilator-associated respiratory infections. J Pure Appl Microbiol.

[17] Jaiswal SR, Gupta S, Kumar RS (2018). Gut colonization with carbapenem-resistant Enterobacteriaceae adversely impacts the outcome in patients with hematological malignancies: results of a prospective surveillance study. Mediterr J Hematol Infect Dis.

[18] Gao B, Li X, Yang F, Chen W, Zhao Y, Bai G, Zhang Z (2019). Molecular epidemiology and risk factors of ventilator-associated pneumonia infection caused by carbapenem-resistant Enterobacteriaceae. Front Pharmacol.

[19] Khater ES, Abdo KM (2022). Detection of carbapenem-resistant Pseudomonas aeruginosa in tertiary care hospital in Saudi Arabia. Microbes and Infectious Diseases.

[20] Cheemala SS, Vara A, Lakshmi MS, Pradhan S, Kalyani K (2023). Phenotypic detection of carbapenemase production in Gram negative bacilli from clinical isolates in a tertiary care hospital in Telangana. J Pure Appl Microbiol.

[21] Vamsi SK, Moorthy RS, Hemiliamma MN, Chandra Reddy RB, Chanderakant DJ, Sirikonda S (2022). Phenotypic and genotypic detection of carbapenemase production among gram negative bacteria isolated from hospital acquired infections. Saudi Med J.

[22] Rajkumar S, Sistla S, Manoharan M (2017). Prevalence and genetic mechanisms of antimicrobial resistance in Staphylococcus species: a multicentre report of the indian council of medical research antimicrobial resistance surveillance network. Indian J Med Microbiol.

